# A model of chronic enthesitis and new bone formation characterized by multimodal imaging

**DOI:** 10.1242/dmm.034041

**Published:** 2018-08-30

**Authors:** Christine Czegley, Clarissa Gillmann, Christine Schauer, Lisa Seyler, Christiane Reinwald, Madelaine Hahn, Michael Uder, Katja Jochmann, Elisabeth Naschberger, Michael Stock, Georg Schett, Tobias Bäuerle, Markus H. Hoffmann

**Affiliations:** 1 Department of Internal Medicine 3, Friedrich-Alexander-University Erlangen-Nürnberg (FAU) and Universitätsklinikum Erlangen, 91054, Erlangen, Germany; 2 Institute of Radiology, Friedrich-Alexander-University Erlangen-Nürnberg (FAU) and Universitätsklinikum Erlangen, 91054, Erlangen, Germany; 3 Department of Developmental Biology, Centre of Medical Biotechnology, Faculty of Biology, University of Duisburg-Essen, 45117, Essen, Germany; 4 Division of Molecular and Experimental Surgery, Department of Surgery, Friedrich-Alexander-University Erlangen-Nürnberg (FAU) and Universitätsklinikum Erlangen, 91054, Erlangen, Germany

**Keywords:** Enthesitis, Gout, Mouse model, New bone formation, Spondyloarthropathy

## Abstract

Enthesitis is a key feature of several different rheumatic diseases. Its pathophysiology is only partially known due to the lack of access to human tissue and the shortage of reliable animal models for enthesitis. Here, we aimed to develop a model that mimics the effector phase of enthesitis and reliably leads to inflammation and new bone formation. Enthesitis was induced by local injection of monosodium urate (MSU) crystals into the metatarsal entheses of wild-type (WT) or oxidative-burst-deficient (Ncf1**) mice. Quantitative variables of inflammation (edema, swelling) and vascularization (tissue perfusion) were assessed by magnetic resonance imaging (MRI), bone-forming activity by [^18^F]-fluoride positron emission tomography (PET), and destruction of cortical bone and new bone formation by computed tomography (CT). Non-invasive imaging was validated by histochemical and histomorphometric analysis. While injection of MSU crystals into WT mice triggered transient mild enthesitis with no new bone formation, Ncf1** mice developed chronic enthesitis accompanied by massive enthesiophytes. In MRI, inflammation and blood flow in the entheses were chronically increased, while PET/CT showed osteoproliferation with enthesiophyte formation. Histochemical analyses showed chronic inflammation, increased vascularization, osteoclast differentiation and bone deposition in the affected entheseal sites. Herein we describe a fast and reliable effector model of chronic enthesitis, which is characterized by a combination of inflammation, vascularization and new bone formation. This model will help to disentangle the molecular pathways involved in the effector phase of enthesitis.

## Abstract (precis)

**Summary:** The authors introduce a new and reliable enthesitis model triggered by innate immune cells and establish multimodal imaging for dynamically assessing inflammation, tissue perfusion and bone remodelling *in vivo.*

## INTRODUCTION

Inflammation of the entheses is associated with excessive bone formation (Schett et al., 2017; Kehl et al., 2016), contrasting the usual catabolic effects of inflammation on bone (Matzelle et al., 2012). New bone formation is insufficiently defined due to difficulties in retrieving tissue from human entheses and the paucity of fast and reliable models that mimic new bone formation in conjunction with enthesitis. In addition, the instruments to reliably measure local bone formation are not well developed, with histopathology as the only exception. Thus, studying new bone formation in the context of enthesitis is still complicated. Developing new models and instruments in this context is urgently required, since specific forms of rheumatic diseases, such as psoriatic arthritis and spondyloarthritis, are characterized by inflammation and new bone formation at the entheses (Ritchlin et al., 2017; McGonagle et al., 2001; Simon et al., 2016; Lories and McInnes, 2012). In such context, new bone formation occurs very rapidly and in part resembles the process of fracture repair with modelling of new bone (Van Mechelen et al., 2017).

Enthesitis is characterized by mechanical triggers leading to local activation of innate immunity accompanied by the influx of neutrophils to the affected sites (Schett et al., 2017; Sherlock et al., 2012). Hence, activation of local neutrophil influx seems a powerful strategy to unleash bone remodelling at enthesial sites and to develop a model to mimic the effector phase of enthesitis. One potent factor that triggers rapid and robust neutrophil influx to tissue sites are crystals, such as monosodium urate (MSU), which are recognized as danger signals and lead to rapid neutrophil attraction (Maueröder et al., 2015; Schauer et al., 2014; Reinwald et al., 2016). Deposition of MSU crystals at enthesial sites leads to inflammation and new bone formation in patients with gout (Dalbeth et al., 2013, 2012; Pineda et al., 2011).

Based on these observations, we developed and characterized a new effector model of enthesitis. By injection of MSU crystals at enthesial sites of *Ncf1* mutant mice [Ncf1** (Sareila et al., 2013; Huang et al., 2000)], we triggered a rapid and consistent bony response with robust new bone formation. The *Ncf1* mutation impairs translocation of the Ncf1 (p47^phox^) protein to the membrane, thereby blocking function of the NADPH oxidase 2 (NOX2 complex) (Sareila et al., 2013; Huang et al., 2000), which is required to degrade inflammatory mediators by neutrophil extracellular trap (NET)-associated proteases (Schauer et al., 2014; Reinwald et al., 2016). We used techniques such as magnetic resonance imaging (MRI), computed tomography (CT) and positron emission tomography (PET) as they allow the quantification and dynamic assessment of new bone formation during enthesitis in a living organism. These approaches were validated by hitherto existing standard conventional methods such as histological analysis.

## RESULTS

### Injection of MSU crystals elicits enthesitis and new bone formation

**Fig. 1.**
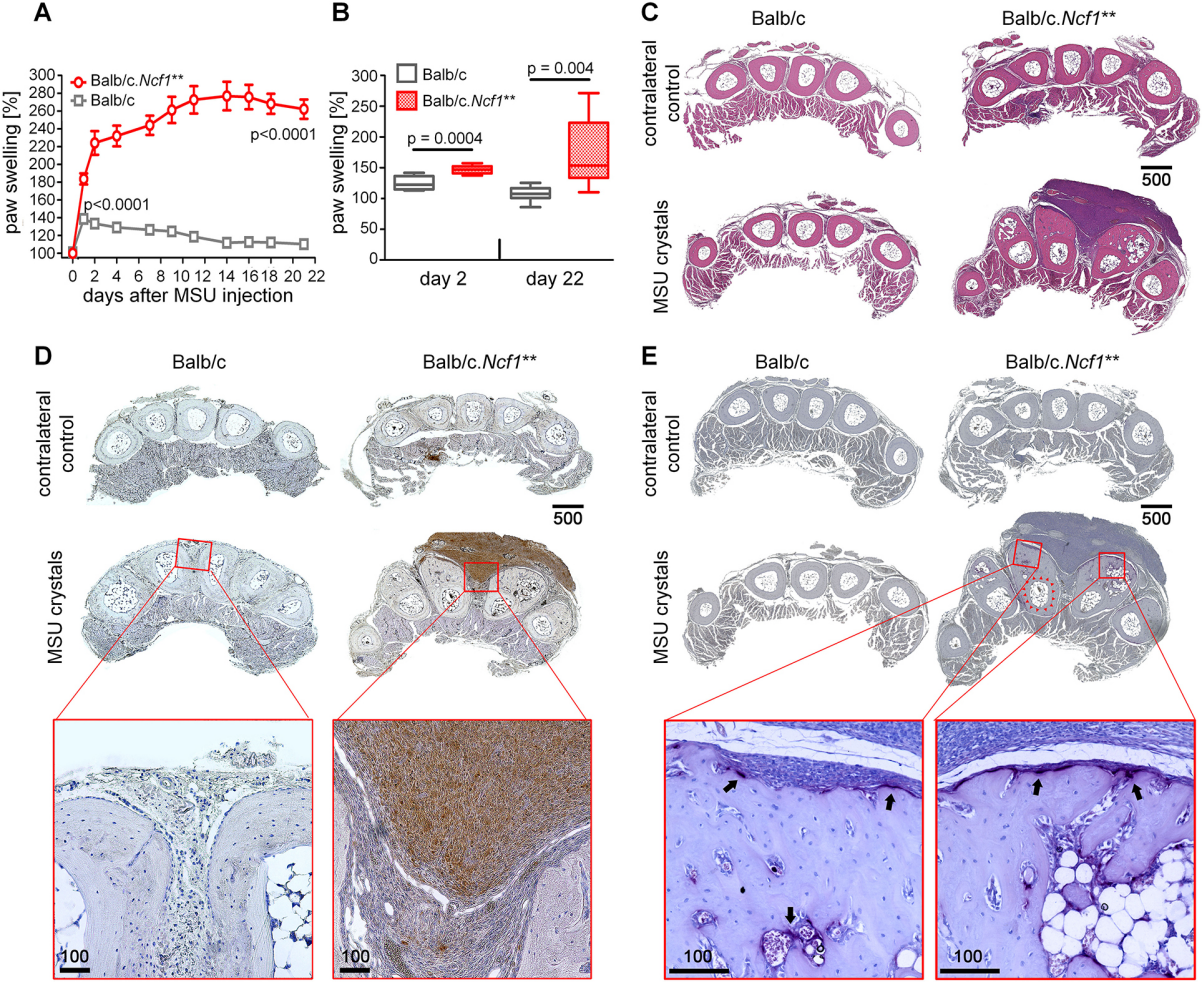
Chronic enthesitis with massive new bone formation after injection of monosodium urate (MSU) crystals in Ncf1** mice. (A,B) Paw swelling elicited by enthesitis after subcutaneous injection of MSU crystals onto the metatarsal ligament insertion sites of BALB/c.Ncf1** and wild-type (WT) BALB/c mice. Plot shows the relative thickness of the MSU-crystal-injected foot normalized to the contralateral PBS-injected foot, as determined by measurement with an electronic caliper (A) or magnetic resonance imaging (MRI) (B). *P*-values in A refer to comparisons at day 1 or day 21, respectively. *P*-values in A and B were calculated by unpaired two-tailed Student's *t*-test. *n*=8-10. H&E stainings (C), immunohistochemical stainings for Ly6G-positive neutrophils (D) and tartrate-resistant acid phosphatase (TRAP) stainings (E) of paws from BALB/c and BALB/c.Ncf1** mice 21 days after injection of MSU crystals or PBS (contralateral control). Figures show representative images chosen from at least five paws. Black arrows in E mark TRAP-positive osteoclasts, red arrowheads indicate the original surface of cortical bone (before new bone formation occurred). Scale bars: 100 µm or 500 µm.

Injection of MSU crystals onto enthesial insertion sites triggered transient mild swelling in wild-type (WT) mice, with no signs of bone disease (Fig. 1A,B). In contrast, when MSU crystals were injected into entheses of Ncf1** mice, sustained swelling developed, as shown by caliper measurements (Fig. 1A) or CT (Fig. 1B). Swelling developed in 100% of mice and its amount and duration was not dependent on the sex of the mouse (not shown). To distinguish between ongoing inflammation and ‘bony swelling’ caused by new bone formation, we performed histological analysis in WT and Ncf1** mice injected with either MSU crystals or vehicle (PBS). Hematoxylin and Eosin (H&E) staining revealed strong inflammation that affected the enthesial sites (Fig. 1C, Fig. S1). Immunohistochemistry showed that vast numbers of Ly6G^+^ neutrophils dominated the inflammatory infiltrate (Fig. 1D). By assessment of tartrate-resistant acid phosphatase (TRAP) activity, we detected bone destruction and large enthesiophytes emerging from the dorsal side of the periosteum of metatarsal bones of Ncf1** but not WT mice (Fig. 1E).

### Non-invasive assessment of bone-forming activity in chronic enthesitis

**Fig. 2.**
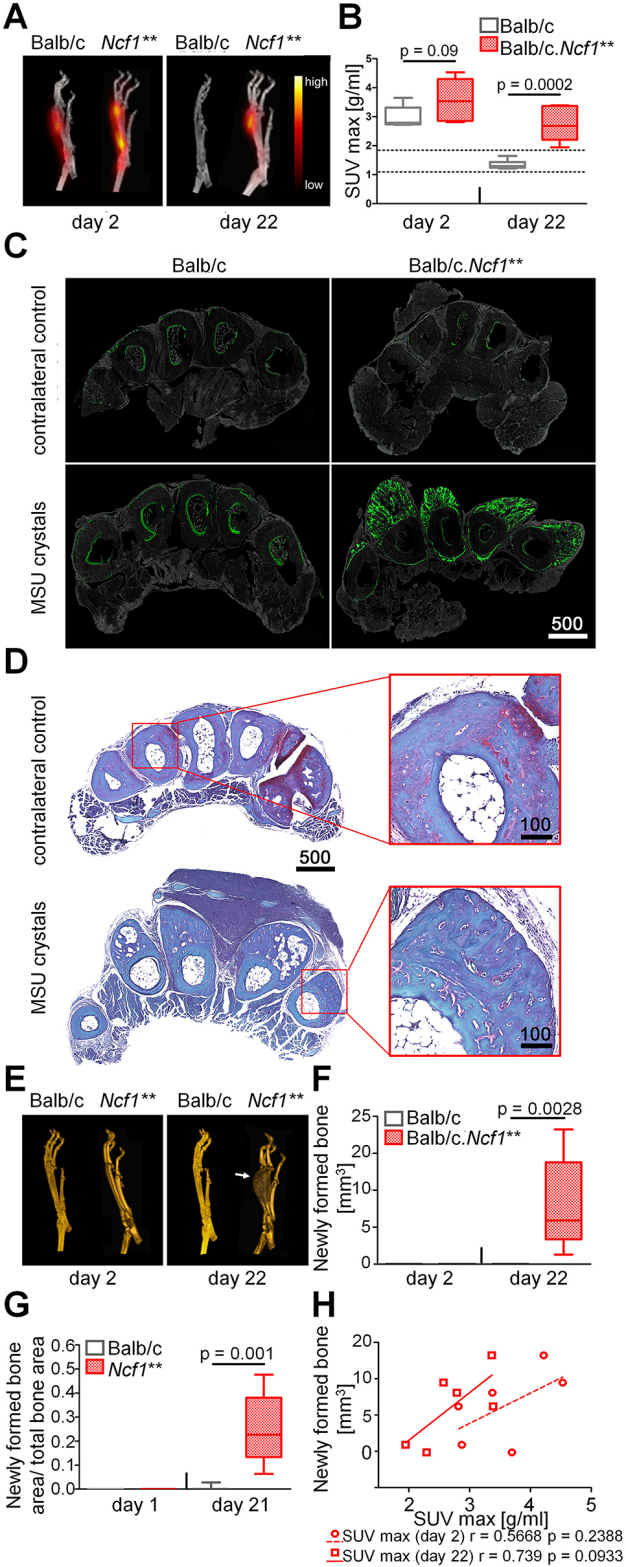
New bone formation in enthesitis of BALB/c and BALB/c.Ncf1** mice as evaluated by PET/CT and histomorphometry. MSU crystals were injected into the metatarsal ligament insertion sites of WT BALB/c and BALB/c.Ncf1** mice, and new bone formation was evaluated 2 and 22 days after by PET/CT using ^18^F or by histomorphometry on TRAP-stained sections. (A) Representative PET images showing the uptake of ^18^F in the metatarsal space (not quantitative) and (B) quantification as maximum standard uptake values (SUV max). (C) Representative images of paws from BALB/c and BALB/c.Ncf1** mice injected weekly with calcein until the end of the experiment 21 days after injection of MSU crystals or PBS (contralateral control). The green calcein signal indicates newly formed and mineralized bone. (D) Safranin-O–Fast-Green stainings of paws from BALB/c.Ncf1** mice 21 days after injection of MSU crystals or PBS (contralateral control). Pictures show representative images from four paws. (E) Representative 3D-volume rendered CT images of MSU-crystal-injected paws and (F) CT quantification of volumes of newly formed bone along the metatarsals. The arrow in E indicates a massive enthesiophyte. (G) Histomorphometrical analysis of newly formed bone in MSU-crystal-induced enthesitis. Boxplots in B, F and G are visualized as follows: horizontal lines show medians, boxes represent interquartile ranges, whiskers display extreme values. *n*=6-11. Dashed lines in B indicate the range of values in non-arthritic (PBS-injected contralateral) paws. (H) Correlation between SUV max at day 2 and 22 days after MSU-crystal injection, and volume of newly formed bone. Scale bars: 100 μm or 500 μm.

The combination of inflammation with new bone formation only in Ncf1** mice suggests rapid pathologic bone turnover in conjunction with chronic but not acute enthesitis. To further assess bone remodelling during transient and chronic enthesitis, we employed PET/CT using [^18^F]-fluoride (Fig. 2A,B, Fig. S2). In WT mice, maximum and mean standard uptake values (SUV max or SUV mean, respectively) in MSU-injected enthesial sites were transiently increased compared to levels seen in PBS-injected sites at day 2, but had reversed to normal levels 22 days after injection. In contrast, SUVs in Ncf1** mice were elevated until 3 weeks after injection of MSU crystals (Fig. 2B, Fig. S2A). Longitudinal assessment of bone-forming activity in individual mice confirmed the stronger decline of bone-forming activity between day 2 and 22 in WT mice (Fig. S2B,C).

To corroborate these results, we performed calcein labelling *in vivo*, which measures the apposition of new bone. Incorporation of calcein was seen at enthesial sites in both WT and Ncf1** animals at day 3 after MSU crystal injection (Fig. S3). In Ncf1** mice, however, the area of bone-forming activity was more extended alongside the periosteal bone surface (Fig. S3B). Furthermore, calcein incorporation in Ncf1** mice was sustained over time and was found at the top of and also within the newly formed bony spurs when calcein was injected weekly until day 21 (Fig. 2C). These results suggest that increased osteoblast-mediated bone formation is associated with inflammation during chronic enthesitis.

To investigate which process leads to the formation of metatarsal enthesiophytes, we employed Safranin-O/Fast-Green staining (Fig. 2D) and collagen-X staining (Fig. S4) on sections of Ncf1** mice with established enthesiophytes. Safranin-O staining revealed that a cartilaginous cap with hypertrophic chondrocytes is completely missing in the bony structures that arise after injection of MSU crystals in Ncf1** mice (Fig. 2D). This was confirmed by a combination of collagen-I and collagen-X staining (Fig. S4): whereas the enthesiophytes contain collagen I, which is expressed in bone, they are largely devoid of collagen X, which is expressed in cartilage. Together with the calcein staining shown in Fig. 2C, these results suggest that an intramembranous ossification process is causing the emergence of metatarsal enthesiophytes in Ncf1** mice.

### Non-invasive assessment of enthesiophytes associated with chronic enthesitis

Quantification of newly formed bone mass by CT (Fig. 2E,F) or histomorphometry (Fig. 2G) revealed that transient enthesitis in WT mice did not result in the formation of enthesiophytes. In contrast, enthesiophytes could readily be detected by both CT and histomorphometry after chronic enthesitis in Ncf1** mice. At the individual level, the intensity of the signal for new bone formation in PET/CT and the size of newly formed enthesiophytes assessed by histology in Ncf1** mice were correlated (Fig. 2H).

### Concomitant catabolic bone changes during enthesitis

**Fig. 3.**
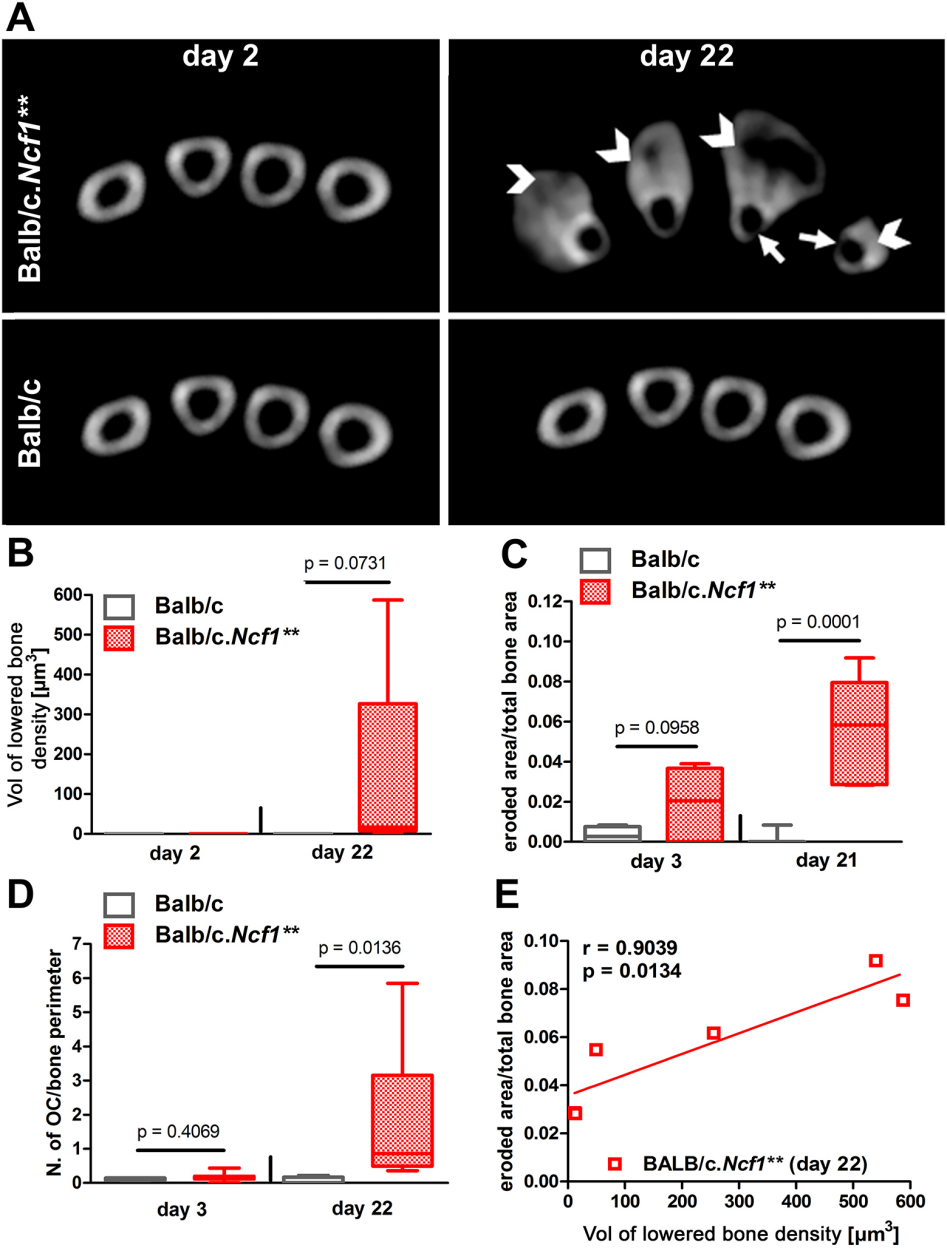
Bone destruction during enthesitis in BALB/c and BALB/c.Ncf1** mice as assessed by CT and histomorphometry. (A) Representative CT images showing the cortical bone and enthesophytes during MSU-crystal-induced enthesitis. Arrows point at lesions where bone density is severely decreased, arrowheads to newly formed bone. (B) Volumes of areas of lowered bone density in the cortical bone as evaluated by CT. (C) Histomorphological analysis of eroded bone and (D) number of TRAP^+^ osteoclasts (OCs) in MSU-crystal-injected paws from BALB/c WT and BALB/c.Ncf1** mice. Horizontal lines show medians, boxes represent interquartile ranges and whiskers display extreme values. *n*=10 (B) or 4-7 (C,D), respectively. (E) Correlation analysis between erosions of the cortical bone measured by CT and histomorphometry.

Bone destruction and new bone formation are often linked in the context of enthesitis. Also in this model, TRAP staining showed long-lasting activity of osteoclasts predominantly in newly formed bone resembling intensive remodelling (Fig. 1E). To quantify these processes, we used CT and histomorphological analysis (Fig. 3). Areas of erosions were detected at late stages of chronic enthesitis of Ncf1** mice using CT analysis and were found in the enthesiophytes as well as in the original cortical bone layer (Fig. 3A,B). Histomorphometry was able to spot erosions already in early disease (day 3), which resolved in WT mice but further increased in chronic enthesitis (Fig. 3C). Also, the numbers of osteoclasts (TRAP-positive multinucleated cells) were strongly increased in chronic enthesitis (Fig. 3D). Destructive changes observed by CT (areas of decreased bone density in cortical bone) and by histomorphometry (eroded area/total tissue area) were positively and significantly correlated (Fig. 3E), suggesting that non-invasive CT imaging reflects the changes observed in the histological analysis.

### Non-invasive assessment of inflammation during enthesitis

**Fig. 4.**
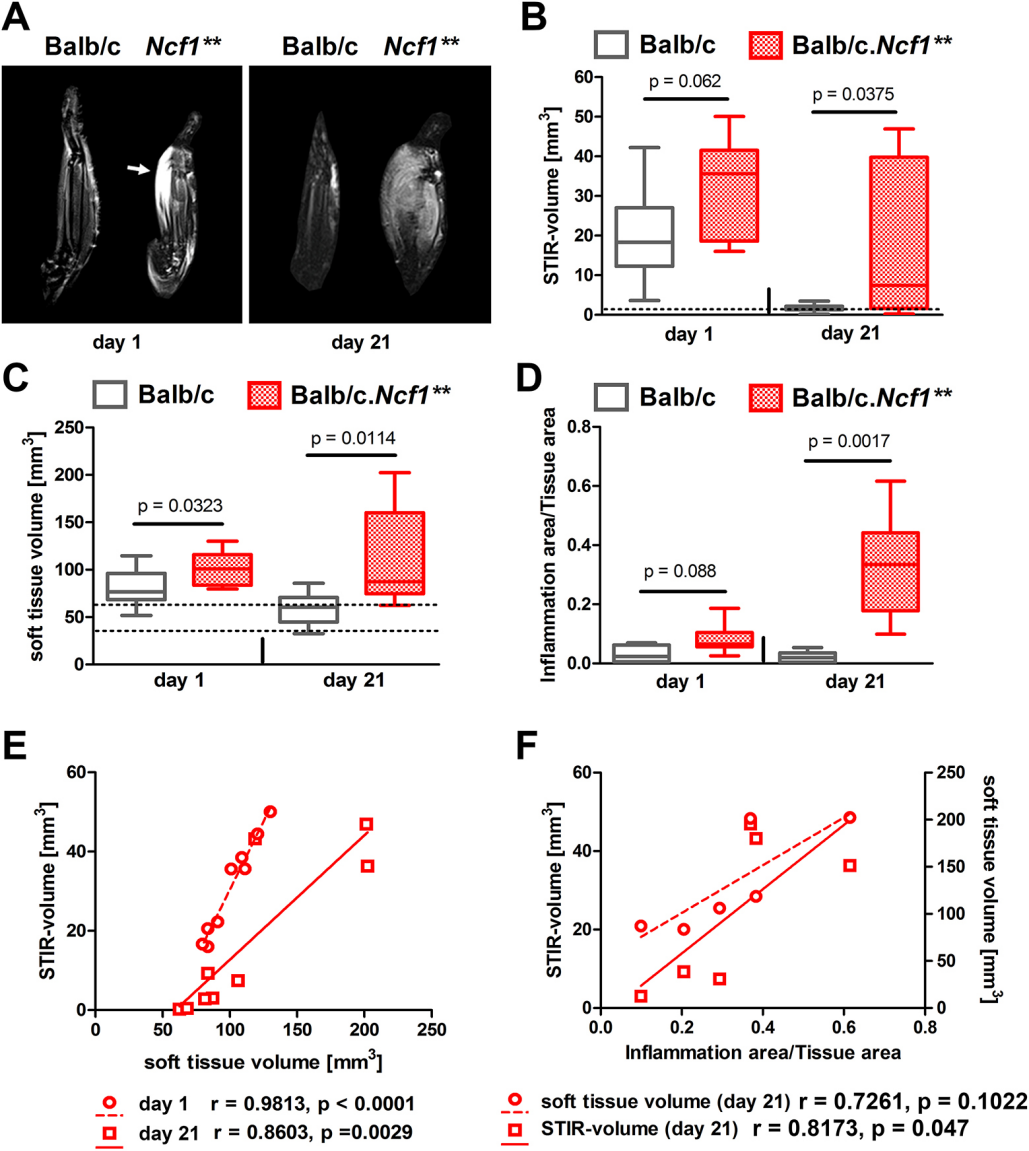
Inflammatory changes during enthesitis in BALB/c and BALB/c.Ncf1** mice as assessed by MRI. MSU crystals were injected onto the metatarsal enthesial insertion sites of BALB/c WT and BALB/c.Ncf1** mice. Paws were evaluated 1 or 21 days after by MRI. (A) Free water content visualized as hyperintense areas (arrow) on representative short tau inversion recovery (STIR) MRI images. (B) Volume of hyperintense areas (STIR volume) and (C) soft-tissue volume, as calculated from MRI. Mice were then sacrificed and areas of inflammatory infiltrates were histomorphometrically analyzed on H&E-stained paw sections (D). Horizontal lines show medians, boxes represent interquartile ranges and whiskers display extreme values. *n*=9-10. Dashed lines indicate the range of values in non-arthritic (PBS-injected contralateral) paws. *P*-values were calculated using unpaired two-tailed Student's *t*-test. (E,F) Correlation of MRI-assessed features of inflammation (STIR volume and soft-tissue volume) with each other (E) and with histomorphometric assessment of inflammation (F) in BALB/c.Ncf1** mice.

Paw swelling in the context of chronic enthesitis is caused by both inflammatory and osteogenic mechanisms. These processes cannot be distinguished by measurement of paw thickness. We therefore employed MRI to also quantify inflammation in chronic enthesitis (Fig. 4A-C). We measured bound tissue water [Fig. 4A,B, visualized by short-tau inversion recovery (STIR)] and soft-tissue volume (Fig. 4C, determined on morphological sequences). Similar to new bone formation, these variables were elevated shortly after induction of enthesitis in WT mice but had normalized at day 21. In contrast, in Ncf1** mice, the elevations remained sustained, which results in significant differences of inflammation between WT and Ncf1** mice at day 21. In addition, a trend to increased inflammation in Ncf1** mice was also observed already at day 1 (Fig. 4B,C). The same course was found when using histomorphometrical analysis of paw sections (Fig. 4D). MRI variables of inflammation (soft-tissue volume and STIR volume) were strongly correlated to each other (Fig. 4E). When correlation between inflammatory variables quantified by MRI and histology was performed, STIR volume reached the level of statistical significance, while soft-tissue volume showed a strong trend (Fig. 4F).

### Non-invasive assessment of vascularization during enthesitis

**Fig. 5.**
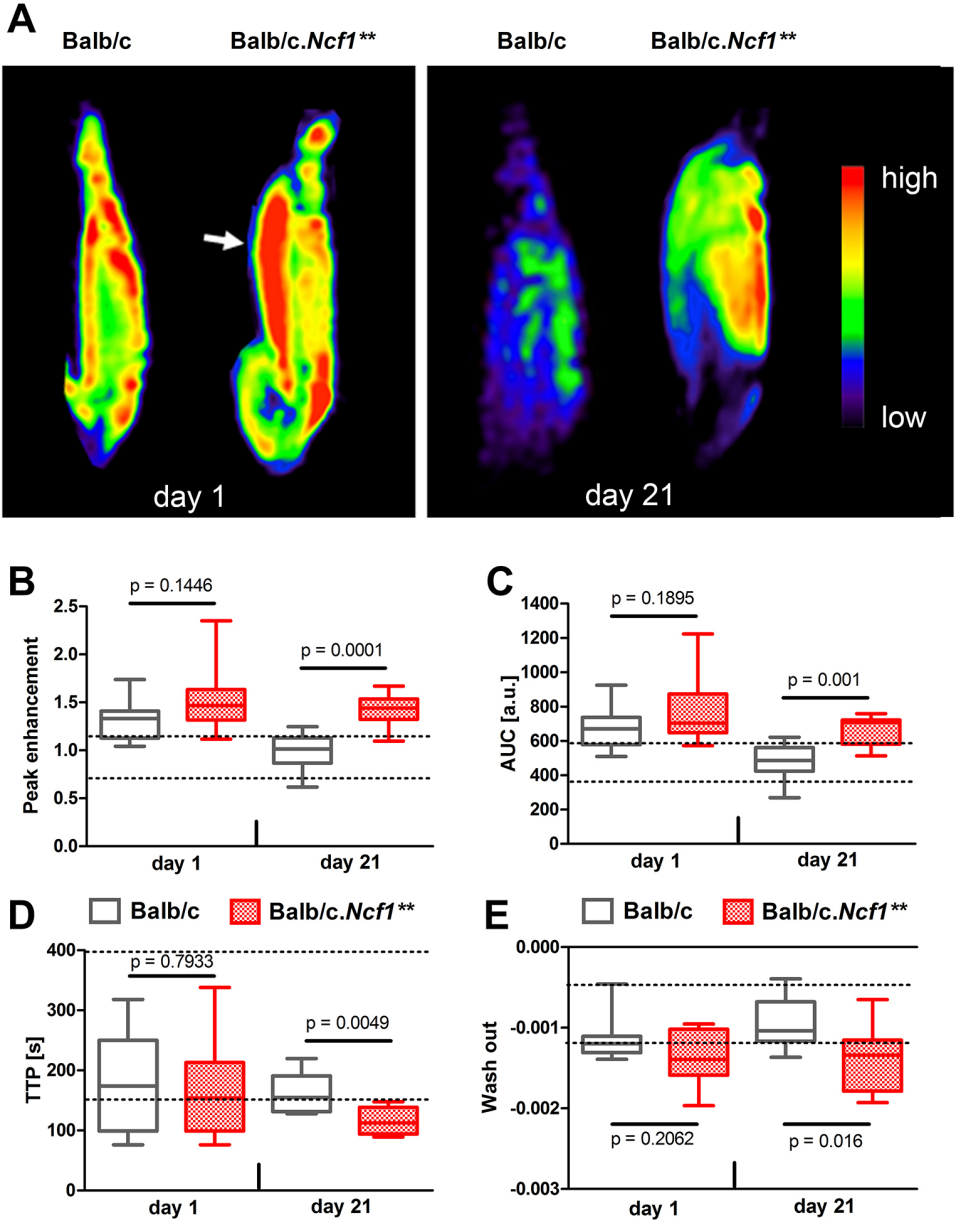
**Increased blood flow and enhanced vascularization in enthesitis.** MSU crystals were injected into the enthesial insertion sites of BALB/c WT and BALB/c.Ncf1** mice. Paws were evaluated by MRI after 1 or 21 days. (A) Colour-coded maps of DCE-MRI depict the distribution of the intravenously injected MR contrast agent (not quantitative). Areas with high blood volume are displayed in red (arrow). From DCE-MRI data, peak enhancement (B), area under the curve (AUC, C), time to peak (TTP, D) and wash out (E) were calculated. Horizontal lines show medians, boxes represent interquartile ranges and whiskers display extreme values. *n*=8-9. Dashed lines indicate the range of values in non-arthritic (PBS-injected contralateral) paws. *P*-values were calculated using unpaired two-tailed Student's *t*-test.

Angiogenesis is an essential feature of enthesitis. For assessing vascularization in spurious and chronic enthesitis, we performed dynamic contrast-enhanced (DCE)-MRI in MSU-crystal-injected WT and Ncf1** mice, respectively. Four variables of tissue perfusion, namely peak enhancement, area under the curve (AUC), time to peak (TTP) and wash out, showed significant differences between WT and Ncf1** mice at day 21 (Fig. 5A-E). In contrast, blood flow was increased during early enthesitis, with no significant differences between the strains.

To validate local angiogenesis in enthesitis, we performed histological analysis of paw sections from WT and Ncf1** mice 21 days after disease onset (Fig. S5). Vessel walls were labelled with anti-CD31 antibody and proliferating vessels were stained for Ki67. While the numbers of CD31^+^ vessels were not increased in the inflammatory tissue between the metatarsals, we found elevated numbers of proliferating CD31-positive vessels in the cavities of newly formed enthesiophytes of Ncf1** mice during chronic enthesitis. These results indicate that the higher perfusion observed in chronic enthesitis is based on elevated angiogenesis in bone tissue rather than angiogenesis directly in the inflammatory infiltrate.

## DISCUSSION

Herein we present a reliable, fast and simple model of chronic enthesitis, which combines inflammation and new bone formation. The model is based on local innate immune cell activation by MSU crystals in a commercially available susceptible mouse strain, which is characterized by impaired resolution of inflammation (Schauer et al., 2014; Reinwald et al., 2016). In this model, which mimics the effector pathway of enthesitis, longstanding inflammation but, even more importantly, profound bone remodelling with enthesiophyte formation occur. Our model shows that aberrant new bone formation is closely related to the chronification of enthesitis. Spurious disease characterized by quick resolution of inflammation and concomitant catabolic and anabolic processes in the bone does not manifest in detectable enthesiophytes.

New bone formation in the context of enthesitis can mimic swelling and makes clinical assessment challenging. Hence, histology is required to disentangle inflammation and bone remodelling. New non-invasive approaches to characterize enthesitis are thus required that allow depiction of the dynamics of enthesitis. The simultaneous presence of inflammation and new bone formation is characteristic for enthesitis. Enthesitis typically starts after forced mechanical stress in healthy individuals but also accompanies diseases such as psoriatic arthritis and spondyloarthritis characterized by activation of the cytokines IL-23 and IL-17 (Schett et al., 2017; Sherlock et al., 2012). These aspects of enthesitis are reflected by the DBA1 model as well as the IL-23 mini-circle model, respectively, which both show signs of enthesial inflammation (Sherlock et al., 2012; Lories, 2004). While such models aim to imitate the triggers of enthesitis, effector models of enthesitis are sparse. Jacques and colleagues have described a T-cell-independent model of enthesitis and new bone formation that relies on weight bearing (Jacques et al., 2014). Clinical observations suggested that crystal deposition, in particular during gout, can also effectively trigger enthesitis and new bone formation (Dalbeth et al., 2013, 2012; Pineda et al., 2011). In particular, metatarsal entheses are subject to considerable mechanical stress and therefore are characteristic sites for MSU crystal apposition, inflammation and bone destruction (Dalbeth et al., 2015). While the triggers for enthesitis may be different, the effector pathways appear remarkably conserved, involving neutrophil activation, angiogenesis and bone formation, and therefore deserve more intensive studies, including appropriate modelling.

Multimodal imaging is an alternative to histology and can also be used in a longitudinal setting. By combining CT, MRI and PET, complementary information on morphological, functional and metabolic levels can be acquired *in vivo* (Beckers et al., 2004; Elzinga et al., 2011; Kundu-Raychaudhuri et al., 2014). MRI detects morphological information on tissue edema or swelling due to its excellent soft-tissue contrast. Furthermore, from DCE-MRI, quantitative data for perfusion and vessel permeability can be obtained. PET can assess metabolic processes such as bone formation after administration of positron emitters such as [^18^F]-fluoride, and CT adds to the morphological information on bone structure. Previous studies have shown that the PET tracers fluorodeoxyglucose ([^18^F]-FDG) and [^11^C]-(R)PK11195 reflect synovitis in human and animal models of arthritis (Kundu-Raychaudhuri et al., 2014; Bruijnen et al., 2012). [^18^F]-fluoride, which targets bone formation rather than inflammation, has been used to assess activity of ankylosing spondylitis and arthritis (Bruijnen et al., 2012; Irmler et al., 2014). [^18^F]-fluoride uptake into bone reflects primarily regional osteoblastic activity because of uptake of the tracer into hydroxyapatite crystals, leading to formation of fluoroapatite at sites of bone formation (Cook and Fogelman, 2001). In this way, PET/CT offers the opportunity to quantitatively assess new bone formation during enthesitis.

DCE-MRI also revealed increased local perfusion in early and late chronic enthesitis. New blood vessels in inflamed tissue are formed from existing blood vessels rather than being built *de novo* or being de-differentiated from other cell types. New vessels sprout from neighbouring tissue after the occurrence of hypoxia, often along a gradient of stimulants such as vascular endothelial growth factor (VEGF). Vascularization is apparent very early after initiation of inflammation and plateaus after 7 days (Ezaki et al., 2001; Cursiefen et al., 2006). Interestingly, in chronic enthesitis the majority of proliferating CD31^+^Ki67^+^ blood vessels was found in the bone cavities of newly formed enthesiophytes. These findings indicate that the bone marrow may be absolutely essential for new bone formation in enthesitis by providing sufficient nutritional support through angiogenesis.

In conclusion, we present a novel model of enthesitis, combining inflammation and new bone formation. We characterized this model in detail by using histology as well as longitudinal multi-modal imaging assessing inflammation, angiogenesis, new bone formation and enthesiophyte growth. Our results show that inflammation and new bone formation are tightly interconnected. This enthesitis model and the assessment tools that we have developed in conjunction with this model will facilitate enthesitis research in the future.

## MATERIALS AND METHODS

### Mice

Mice with a mutated *Ncf1* gene (*Ncf ^m1j/m1j^*; denoted as Ncf1**) were kindly provided by Prof. Rikard Holmdahl (Karolinska Institute, Stockholm, Sweden) and have been backcrossed over more than 12 generations to the BALB/c background. Ncf1** mice and WT BALB/c littermates were bred and maintained in-house. Experiments were performed with blind evaluation on 8- to 12-week-old female and male mice. Experimental groups were frequency-matched for sex and age. All animal procedures were in accordance with institutional guidelines on animal welfare and were approved by the local ethical committee of the University Erlangen-Nuremberg (Regierung von Unterfranken, Würzburg, Germany). Mice were allocated randomly into groups by a computer-based random number generator (http://www.randomizer.org) so that each cage contained animals of every group to compensate for possible cage effects. Power analysis was performed based on effect size estimates from previous experiments (data not shown).

### MSU-crystal-induced enthesitis

MSU crystals were produced as previously described (Schauer et al., 2014). For induction of enthesitis, 1.5 mg MSU crystals suspended in 70 µl PBS were injected into the dorsal sides of the distal metatarsal bone of BALB/c and BALB/c.Ncf1** mice at sites of enthesial insertions. As a control, the respective contralateral side was injected with 70 µl PBS. Paw thickness was measured with an electronic caliper at the indicated time points.

### MRI imaging

*In vivo* imaging by MRI of the hind paws was performed at days 1 and 21 after injection of MSU crystals. MRI was performed on a preclinical 7 T ultra-high-field scanner (ClinScan 70/30, Bruker, Ettlingen, Germany) using a dedicated surface coil (Bruker), on which the hind paws were placed. The imaging protocol included a T1-weighted spin echo sequence [repetition time (TR)/echo time (TE): 556/9.0 ms, inversion time (TI): 140 ms, field of view (FoV): 35×75, matrix: 448×448, in-plane resolution (res): 0.078×0.078 mm, slice thickness: 0.7 mm, averages (av): 2, acquisition time (TA): 5:29 min], a STIR sequence (TR/TE: 4110/33 ms, FoV: 35×100, matrix: 320×320, res: 0.109×0.109 mm, slice thickness: 0.7 mm, av: 1, TA: 6:26 min) and a multi-echo spin echo sequence (TR: 2710 ms, TEs: 10.2-71.4 ms in 7 intervals, FoV: 35×75, matrix: 320×320, res: 0.109×0.109 mm, slice thickness: 1 mm, av: 1, TA: 10:53 min). DCE-MRI was performed using a fast low-angle shot (FLASH) sequence with the following parameters: TR/TE: 2.47/0.88 ms, flip angle: 25°, FoV: 35×75, matrix: 128×128, res: 0.273×0.273 mm, slice thickness: 0.7 mm, av: 1, measurements: 100, TA: 10:52 min. After 30 s baseline, 0.1 mmol/kg Gd-DTPA (Magnevist, Schering, Germany) was infused intravenously over a time period of 10 s via a tail vein catheter. For post-processing of the acquired data of the multi-echo spin echo-sequence, voxel-based 3D-parameter maps of T2-relaxation times were calculated for each paw (SyngoVia software, Siemens, Erlangen, Germany).

### MRI analysis

For MRI analysis, the volume of the metatarsal space of each hind paw was segmented on T1-weighted morphologic images. The segmentation mask was transferred to the T2 relaxation time map, and the mean T2 time in the segmented volume was derived. In the same way, however, including only soft-tissue volume and excluding bones, volume and T2 time of soft tissue were derived. Inflammation is commonly associated with edema and increased water incorporation, visible as hyperintense areas on STIR images. The volume of edema (bound tissue water) in the metatarsal space was therefore quantified by segmenting all voxels in the metatarsal space with signal intensity above 300 a.u. on STIR images.

DCE-MRI data were analyzed using a Horos DICOM Viewer in conjunction with the DCE plugin (V 2.2, K. Sung, UCLA). For each animal, the metatarsal space of the left and right hind paw was delineated on one representative slice of the image stack. DCE curves were calculated and, from these, the variables area under the curve, peak enhancement, time to peak and washout were derived using a self-written software script (DCE plugin, Osirix DICOM viewer).

### PET/CT imaging

*In vivo* imaging by PET/CT of the hind paws was performed at days 2 and 22 using a preclinical hybrid scanner (Inveon, Siemens, Erlangen, Germany). A total of 4.5 MBq sodium fluoride labelled with fluorine-18 (^18^F-NaF) were intravenously injected into the animals 25 min prior to imaging. CT imaging was performed with the following settings: tube voltage: 80 kV, tube current: 500 µA, acquisition: step-and-shoot, rotation: full, settle time: 500 ms, projections: 180, exposure time: 1100 ms, binning: 2×2, charge-coupled device (CCD) size: 1664×1664 px, FoV: 40×40 mm, effective pixel size: 49 µm, scan time: 6 min. After CT, PET images were acquired for 15 min. CT images were reconstructed with a Feldkamp algorithm and a Shepp-Logan filter. Reconstruction of PET images was performed with the system manufacturer's implementation of a 3D-ordered subsets expectation maximization (OSEM) and a shifted Poisson maximum a posterior (SP-MAP) algorithm with the following settings: matrix size of reconstructed image: 128×128, pixel size of reconstructed image: 0.77×0.77×0.79 mm, image zoom: 1, OSEM 3D iteration number: 2, SP-MAP iteration number: 18, SP-MAP target resolution: 1.5 mm, attenuation correction: from CT image, scatter correction: none.

### PET/CT analysis

For CT image analysis, window level was set to 3759, window width to 5553. The visible newly formed bone along the metatarsals was segmented and its volume was determined. Osteolytic lesions in the metatarsal space were segmented with threshold values between 1000 and 2500 Hounsfield units. PET data were analyzed using Inveon acquisition software (Siemens, Erlangen, Germany). For each animal, the left and right hind paws were segmented, and mean and maximum activities in the segmented volumes were determined. Mean and maximum standard uptake values (SUV mean and SUV max) were calculated by dividing the obtained mean and maximum activities by the injected activity and multiplying the result by the weight of the animal. CT and MR image analysis was performed using Osirix Dicom Viewer (Aycan Osirix, USA) in conjunction with Chimaera's segmentation tool (Chimaera GmbH, Erlangen, Germany).

### Histology and immunohistochemistry

Paws were fixed overnight in 4% formalin, decalcified with EDTA and embedded in paraffin. For histology as well as immunohistology, paraffin-embedded tissue sections were deparaffinized in xylol (Merck Chemicals) and rehydrated in a descending ethanol series. For the assessment of inflammation, sections were stained with H&E. TRAP staining was used to evaluate the number of osteoclasts and bone destruction. Furthermore, slides were stained with Safranin-O–Fast-Green for the evaluation of the proteoglycan contents. Slides were analyzed on a Zeiss AxioLab.A1 microscope equipped with a digital analysis system (OsteoMeasure™, Osteometrics).

For analysis of angiogenesis or neutrophils in paw sections by immunohistochemistry, deparaffinized ethanol-dehydrated tissue sections were heated in a water bath (95°C, 20 min) for epitope retrieval using an antigen retrieval solution (pH 9.0; TRS9; Dako) and blocked with 10% donkey normal serum for 1 h at room temperature or 0.2% BSA in PBS, respectively. Neutrophil staining further required endogenic oxidase blockade with 3% hydrogen peroxide for 5 min. For analysis of angiogenesis, sections were stained with a monoclonal rat anti-mouse CD31 antibody (Dianova; clone SZ31; 5 µg/ml) and a rabbit anti-Ki67 antibody (Abcam, clone SP6; diluted 1:50) for 1 h at room temperature and subsequently incubated for 45 min with an Alexa-Fluor-488-conjugated donkey anti-rat IgG antibody (Thermo Fisher Scientific, cat. # A-21208, 4 µg/ml) or an Alexa-Fluor-546-conjugated donkey anti-rabbit IgG antibody (Thermo Fisher Scientific, cat. # A10040, 4 µg/ml). Nuclei were stained with DAPI (Dako) and slides were mounted with fluorescence mounting medium (Dako). For staining neutrophils, sections were incubated with rat anti-mouse-Ly6G antibody (BioLegend, cat. #127601, clone 1A8, 1:1000 in blocking buffer) and horseradish peroxidase (HRP)-conjugated goat-anti-rat secondary antibody (Southern Biotech, cat. #3030-05, 1/500 in blocking buffer). The HRP substrate 3,3′-diaminobezidine (Vector Labs DAB-kit #SK4100) was used as substrate for HRP.

Immunohistochemistry for collagen I (Col I) and collagen X (Col X) was performed as previously reported (Eitzinger et al., 2012). For the detection of Col I, rehydrated paraffin sections were blocked with 5% bovine serum albumin (BSA) in PBS prior to incubation with rabbit anti-Col-I antibody (1:500 in 5% BSA/PBS; ab 21286; Abcam) overnight at 4°C. After washing, bound anti-Col-I antibody was detected using the Link-Label IHC Detection System (Biogenex, San Ramon, CA) with biotinylated anti-rabbit IgG, avidin-coupled alkaline phosphatase and Fast Red/Naphtol tablets containing levamisole (Sigma-Aldrich) according to the manufacturers’ instructions. For the detection of Col X, antigen retrieval was performed using testicular hyaluronidase (2 mg/ml in PBS, pH 5.0, H3506; Sigma-Aldrich). Subsequently, Col X immunodetection was performed using protein G sepharose-purified mouse monoclonal anti-Col-X antibody X53 (1:100) (Girkontaite et al., 1996), M.O.M. Basis Kit (BMK-2202; Vector Laboratories), anti-mouse-IgG Link-Label IHC Detection System (Biogenex) and Fast Red/Naphtol (Sigma-Aldrich) according to the manufacturers’ instructions.

### *In vivo* calcein labelling

For detection of early bone formation, mice were injected intraperitoneally with 0.6 mg calcein (Sigma-Aldrich) 7 days prior to and together with MSU crystals challenge. For detection of late changes, mice received calcein injections at day 7, 14 and 21 after MSU crystals. At 3 days after the last injection (day 3 or 24 after MSU challenge), mice were sacrificed, paws were fixed in ethanol and then methanol, embedded in methylmethacrylate and sectioned along the sagittal or coronal plane. Pictures were taken by Zeiss AxioLab.A1 microscope. Mineralizing periosteal surface (surface with a calcein double layer) and total periosteal bone surface were calculated on the OsteoMeasure™ system.

### Statistical analysis

Two-group comparisons were performed using paired or unpaired two-tailed Student's *t*-test with Welch's correction in the case of unequal variance or one sample *t*-test. Outliers within data sets were excluded based on a Grubb's test/extreme studentized deviate (ESD) test for variation from a normal distribution. *P*-values less than 0.05 were considered statistically significant. Computations were performed and charts were produced using GraphPad Prism 5 software.

## Supplementary Material

Supplementary information
