# Supplementary material for: A model of chronic enthesitis and new bone formation characterized by multimodal imaging
Source: Dis Model Mech. 2018 Aug 30;11(9):dmm034041. doi: 10.1242/dmm.034041 (PMC6176995; doi:10.1242/dmm.034041)
Supplement: Supplementary information [file dmm-11-034041-s1.pdf]

SUPPLEMENTARY FIGURES

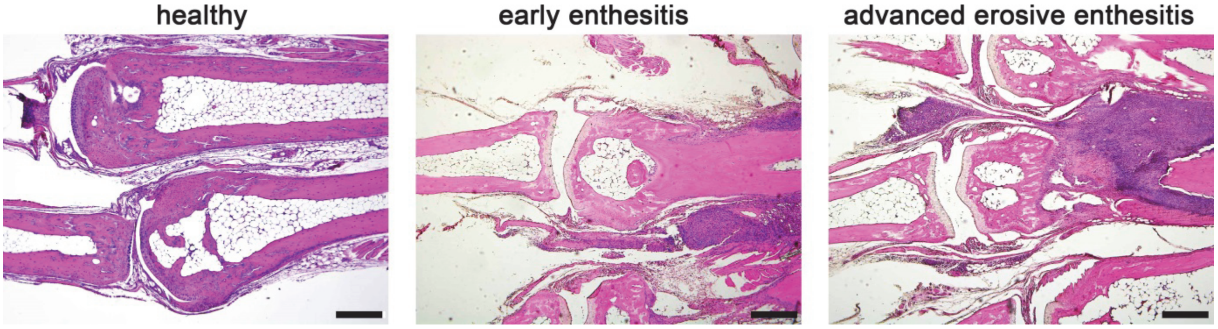

Figure S1: Entheseal inflammation and bone erosion after injection of MSU crystals. Haematoxylin & Eosin stainings of the proximal interphalangeal joints. of a PBS-injected paw (healthy) and an early and advanced erosive stage of MSU crystal-induced enthesitis in a Balb/c.*Ncf1*** mouse. Scale bars, 200 µm

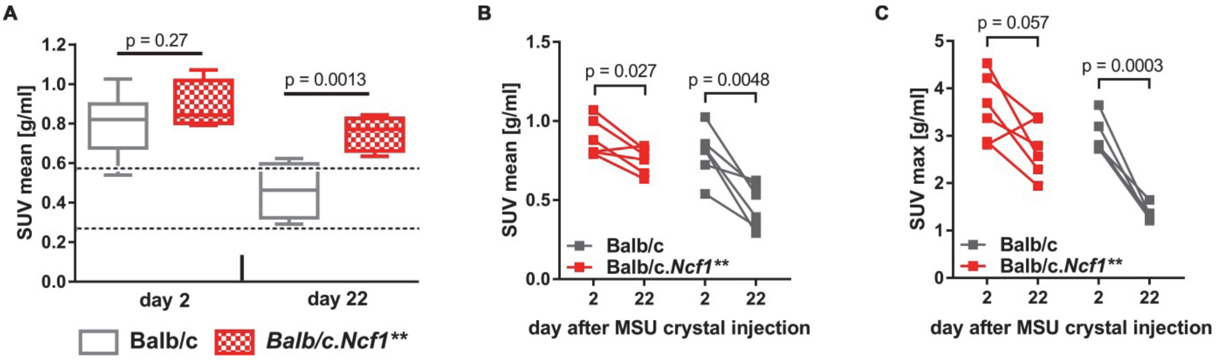

Figure S2: Standard uptake volume of ^{18}F in MSU crystal-induced enthesitis. (A) Quantification of uptake of ^{18}F in the metatarsal space of Balb/c and Balb/c.*Ncf1*** mice early (day 2) and late (day 22) after injection of MSU crystals as mean standard uptake values (SUV mean). Horizontal lines show medians, boxes represent interquartile ranges, whiskers display extreme values. N = 6. Dashed lines indicate range of values in non-arthritic (PBS-injected contralateral) paws. (B, C) Longitudinal follow up of SUV mean and SUV max in individual mice (N = 5). P values were calculated using unpaired (A) or paired (B, C) two-tailed Student´s t-test.

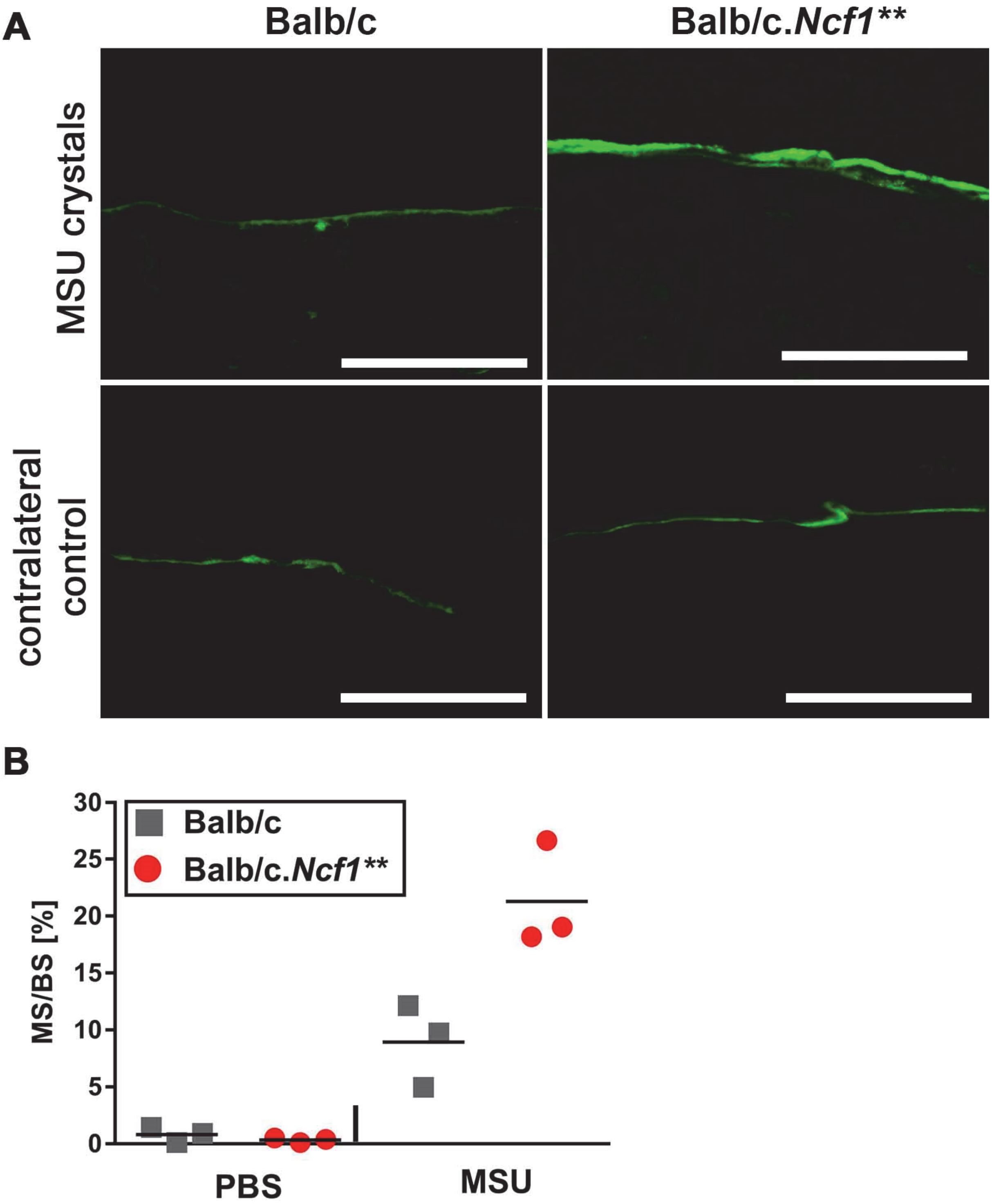

Figure S3: Analysis of new bone formation during early MSU-induced enthesitis by calcein labeling *in vivo*. (A) Representative images of paws from calcein-injected Balb/c and Balb/c.*Ncf1*** mice 72 hours after injection of MSU crystals or PBS (contralateral control). Stronger periosteal calcein apposition and the formation of a double layer is apparent in *Ncf1*** animals. Scale bars, 100 µm. (B) Quantification of periosteal mineralizing surface (MS)/bone surface (BS) in individual mice 72 hours after injection of MSU crystals/PBS. One symbol represents one mouse.

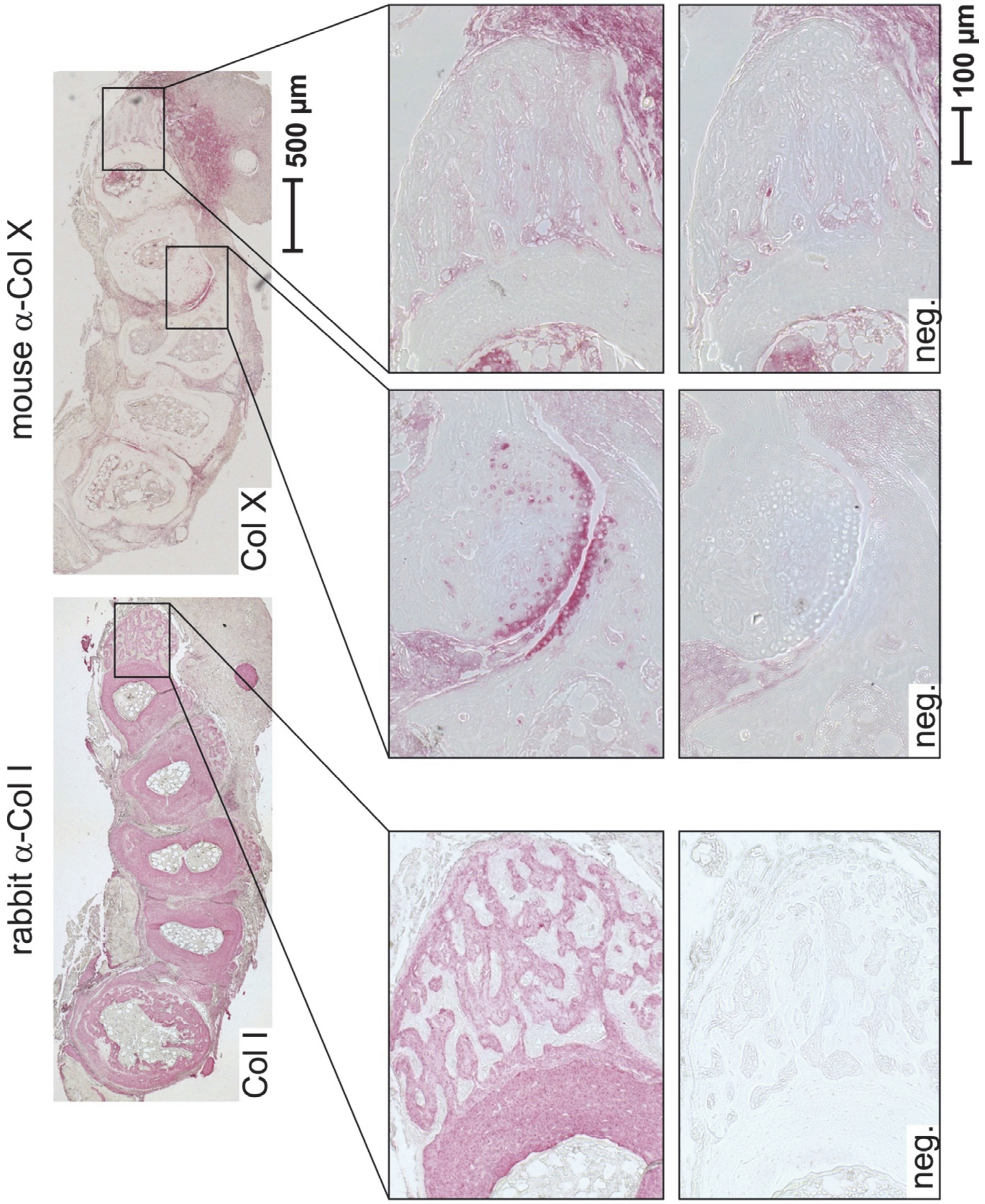

Figure S4: Collagen I and collagen X-staining in established enthesiophytes. Representative images of paw sections from Balb/c.*Ncf1*** mice 21 days after injection of MSU-crystals and stained with antibodies to collagen type I (Col I) and type X (Col X). Col I staining is abundantly expressed in cortical and newly formed bone (left panel). Col X is apparent in the articular cartilage of joints (middle panel), but absent in newly formed enthesiophytes (right panel). As staining controls (neg.), sections were incubated with secondary antibody only.

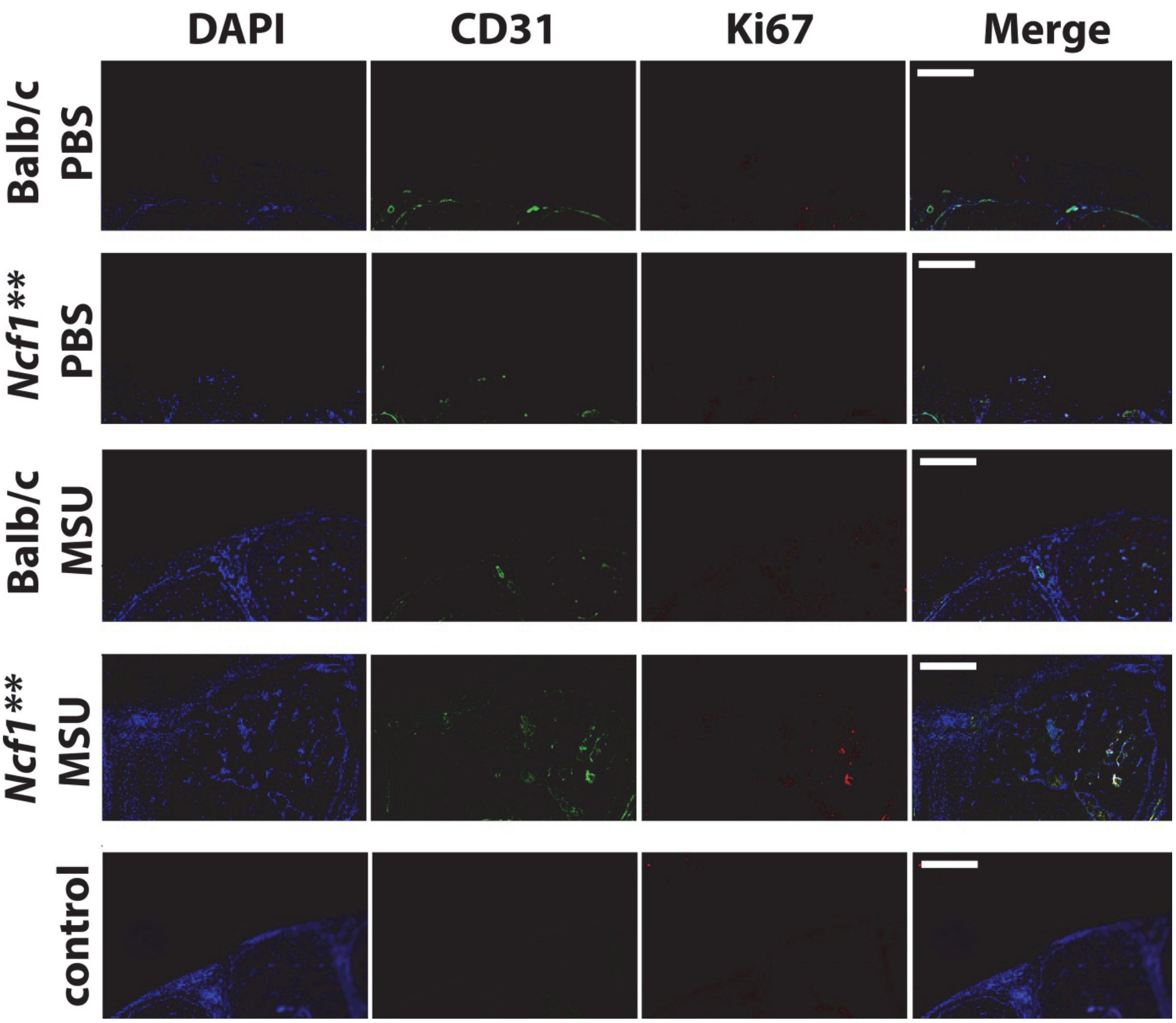

Figure S5: Histological analysis of angiogenesis in MSU crystal-induced enthesitis. Representative images of tissue sections of paws from Balb/c wild type and Balb/c.*Ncf1*** mice stained with antibodies to CD31, Ki67, and with the DNA dye DAPI. Scale bars, 500 µm. Control sections shown are from an MSU crystal-injected wild type mouse and were incubated with secondary, fluorophore-conjugated antibodies and DAPI, but without primary antibodies.
